# Gender differences in general health and happiness: a study on Iranian engineering students

**DOI:** 10.7717/peerj.14339

**Published:** 2022-11-03

**Authors:** Asieh Namazi

**Affiliations:** Department of Physical Education and Sport Science, Iran University of Science and Technology (IUST), Tehran, Tehran, Iran

**Keywords:** Engineering students, General health, Happiness

## Abstract

This article aims to determine the relationship between general health and happiness in male and female engineering students in Iran. The analysis relies on a unique dataset collected through two questionnaires (Oxford Happiness and GHQ28) from a representative sample (*n* = 2,000). Although similar data have been collected quite independently by different communities of researchers, and empirical evidence points to very similar underlying mechanisms, but they did not consider non-normal distribution samples. In this study, data were analyzed by Kruskal–Wallis and Wilcoxon’s signed rank tests. The mean happiness and general health were 42.89 ± 14.84 and 23.76 ± 13.28, respectively. There was a positive and significant relationship between these two variables (*P* ≤ 0.05, r = 0.59). Happiness showed significant differences in males and females, but general health did not. In general, female students enjoyed more health and happiness. This study argues that clarifying the relationship between happiness and health in different genders can help academic authorities and youth planners to pay special attention to this issue and provide particular programs.

## Introduction

The United Nations (UN) General Assembly decided in 2011 to invite all member states to measure happiness to assist policy makers. As a result, positive psychology has become a recognized sub-discipline, with professional learned societies, journals, and postgraduate degrees from universities in the United States, the United Kingdom, Canada, Australia, Denmark, Singapore, and other countries. Numerous books have extolled the value of happiness (Diener & Biswas-Diener, 2002; Dolan, 2014; Graham, 2012; Layard & Layard, 2011; Lyubomirsky, King & Diener, 2005), and life coaching for happiness is a thriving industry. Noddings (2003) in her book, Happiness and Education, argues the importance of happiness in the education system, and believes that it is the duty of education to boost happiness in the community. This attention has, in turn, led to skeptical reactions from many quarters (Steptoe, 2019), and nowhere has the controversy been more significant than in the health arena. There is no one-sided issue in examining the relationship between health and happiness. In other words, this relationship is two-sided. Broadly, the finding of researchers, for example, H Choi, E Diener, J Sim, S Oishi, 2020 (unpublished data) indicate that happiness accounted for associations with various desirable behavioral outcomes such as health behaviors. The results of the study conducted by DiMaria, Peroni & Sarracino (2020) in a sample of 20 European countries confirm that promoting happiness is not only desirable *per se*, but it is conducive to higher productivity and improved countries’ economic performance. Happiness evaluation in the sample of U.S. Army soldiers included both males and females (mean age 29.60 years old) indicated an almost fourfold greater health and high well-being in happier group when the highest *vs* lowest happiness were compared (Lester et al., 2022). Likewise, the results of studies that examined and compared health and happiness in both genders have been contradictory. Some of them showed almost no difference between the happiness of males and females (Diener, Oishi & Lucas, 2003; Fujita, Diener & Sandvik, 1991), and most slight variations are occurred by country of origin or cultural factors (Meisenberg & Woodley, 2015). A study by Francis et al. (1998) in Canada, the United States, the United Kingdom, and Australia reported average happiness scores for girls 32 and boys 42. Also, in Duisburg University Germany (2003), this mean was shown to be 41.6 and 43.1 for boys and girls, respectively. There was not any meaningful difference between them (Francis, Ziebertz & Lewis, 2003). Kamthan et al. (2019) found that male students were happier than females. A cross-sectional survey of undergraduate university students (mean age $20.9\pm 2.8$) from 25 universities in 24 countries across Asia, Africa, and the Americas indicates that the overall mean score of happiness was 13.7 (range 4–20). Generally, the study found that university students from countries in the Caribbean, South America, and sub-Saharan Africa had greater happiness scores than students from countries in North Africa and Asia (Peltzer et al., 2017). (On the happiness scale, the level was low (a score of 0 to 28), medium (a score of 29 to 57), and high (a score of 58 to 87)). Today, community health, especially students as future makers, is taken into consideration. Prevention of complications induced by physiological and psychological diseases has become important. Quality and satisfaction are a priority for young people and student’s life. We agree with Noddings about the duty of education systems. The first step is to determine the current situation of happiness in university and school to achieve this goal. The low ranking of Iran in the global report of happiness (Sachs, Layard & Helliwell, 2018) reflects the negligence of the need for happiness in Iran’s educational system. Joy is not a priority, and there is no plan for the happiness of students not only the educational system does not try to rejoice students, but it also does not provide the conditions for happy programs. On the other hand, there is not enough time for happy programs due to studying and university entrance exam pressure. Schools and universities do not care about students’ self-awareness and identification process. Some students are not interested in their educational orientation, and many are not well aware of their future careers. Lack of valuable skills, creativity, inactive teaching method, *etc*., shows the current situation educational system (Shamshiri & Dastouri, 2018). Psychologically, the happiness is affected by the environment. Iranian engineering students are less delighted compared to other university students because of work conditions. They face much stress on a daily basis that endangers their mental and physical health. Student life, unfamiliarity with the new educational environment, distance from family, incompatibility with other people in the student living environment, socio-economic problems, and lack of welfare facilities are some of the factors that cause psychological problems and discomfort, and eventually, academic failure. Determining the status of happiness in Iranian engineering students was the first goal of this study and then was aimed to find out the relationship between general health and happiness in male and female students and compare the two genders in this regard. The main question of the research was, “Is there any significant difference between general health and happiness of male and female students?” This study is important in universities because it can directly relate to students’ academic achievement. Therefore, such studies will help academic planners and those involved with Youth Affairs, and can be used to improve happiness in universities of Iran and even in other countries.

## Materials and Methods

The present study was descriptive-comparative. A total of 2,000 students of the Iran University of Science and Technology (IUST) in Tehran participated in this study voluntarily. They were verbally informed about the purposes of the study. With guarantees of the participants’ anonymity, informed consent was verbally obtained from all prior to data collection. The Oxford Happiness Questionnaire with 28 items was used to collect the required data. The total score on the scale is from 0 to 87. In addition, the General Health Questionnaire (GHQ) was employed. This questionnaire was developed in 1979 by Goldberg and Hiller and has four sub-scales: (1) physical symptoms, (2) anxiety symptoms, (3) social functioning, and (4) depression symptoms. Kruskal Wallis, Wilcoxon’s signed rank tests, and Tukey’s post-hoc tests were run to analyze the data.Table 1 displays the mean and standard deviation of general health and its sub-scales and happiness in both males and females. Social function, considered equal in both groups, and anxiety have the highest scores. Although females had a higher deviation in total health, they were about two units more than males in mean. On the other hand, the mean of happiness in females was six units more, and the standard deviation was equal in both.

**Table 1:** Mean and standard deviation of health and its subscales and happiness in two group.

Index	Females		Males		Total	
	Mean	SD	Mean	SD	Mean	SD
Physical symptoms (CA)	6.62	$\pm 4.74$	5.22	$\pm 2.92$	6.02	$\pm 4.11$
Anxiety (CB)	6.91	$\pm 5.37$	6.63	$\pm 4.29$	6.79	$\pm 4.92$
Social functioning (CC)	7.94	$\pm 3.48$	7.97	$\pm 3.06$	7.96	$\pm 3.29$
Depression (CD)	3.15	$\pm 4.67$	2.77	$\pm 3.19$	2.99	$\pm 4.09$
Total health (C Total)	24.68	$\pm 15.17$	22.61	$\pm 10.31$	23.76	$\pm 13.28$
Total happiness (D Total)	45.73	$\pm 14.64$	39.06	$\pm 14.37$	42.89	$\pm 18.84$

**Figure 1:**
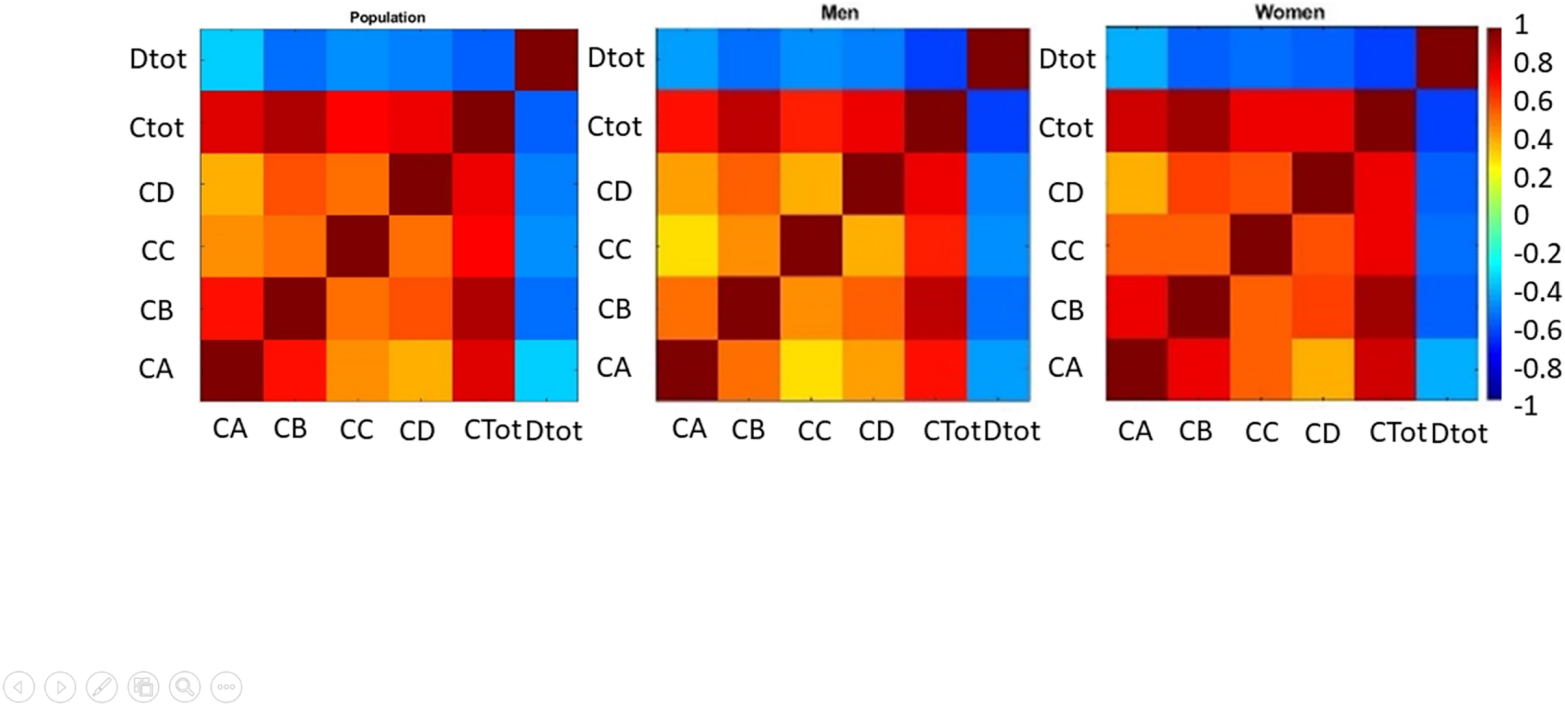
Correlation between total health and its subscales and happiness in the sample. The correlation for all samples shows in left panel, men are shown middle panel, and women are shown in left panel.

In Fig. 1 (left), the strong correlation between physical symptoms and anxiety and its relationship with social functioning was about 0.7 and more than %40, respectively. The highest correlation was between depression and total health, anxiety, and happiness.

Figure 1 shows that in the female group (right), four sub-scales have a high and strong correlation with each other and total health. In the male group (middle), the physical symptoms had little correlation with social function, but this association was not statistically significant (*P* = 0.037) (*r* = 0.2967, *P* = 0.0505). The highest correlation exists between total health and anxiety in both groups. Also, depression correlates with general health, but the relationship between these indices is slightly more robust in females. Happiness has a strong association with the general health and anxiety index. The slightest relationship between happiness and physical signs was reported. The correlation between happiness and all indices except for the social function was high in females, but the relationship between physical signs and happiness was higher in males. The most important correlated factor with happiness in females was depression and anxiety in females and males groups, respectively. There was the least possible association between physical symptoms and social function and depression in boys and girls, respectively. According to the statistical distribution of data, the signed rank test was used for the whole population and female and male groups separately. The most noticeable feature of this test can be considered its unrestricted use for abnormal distributions. This test is used as an equivalent to the T-test for normal distributions.

Table 2 shows a significant difference between health and happiness at the 5% level in all three groups (total, girls, and boys). (The number of *P* represents the probability). Happiness and health in all three groups were completely different so that the reported probabilities are close to zero. In order to better show the results of the signed rank test, it was followed by the Kruskal–Wallis Test. The Kruskal test is a non-parametric equivalent of the one-way ANOVA test. Compared to the ANOVA, which is suitable for normal distributions, the K2 test is used in this test, while the F is used in the ANOVA test.

**Table 2:** Results of the signed rank test for the relationship between total health and happiness totally and separately for the two groups.

Health-Happiness	*P* sign	*P* signed rank	Z value sign	Z value signed rank	Sign	Signed rank
Total	1.158 * 10^−7^	9.819 * 10^−10^	−5.3	−6.1	23	747.5
Males	0.0019	1.256 * 10^−4^	–	−3.8	11	155.5
Females	1.312 * 10^−5^	2.313 * 10^−6^	–	−4.7	12	232

**Figure 2:**
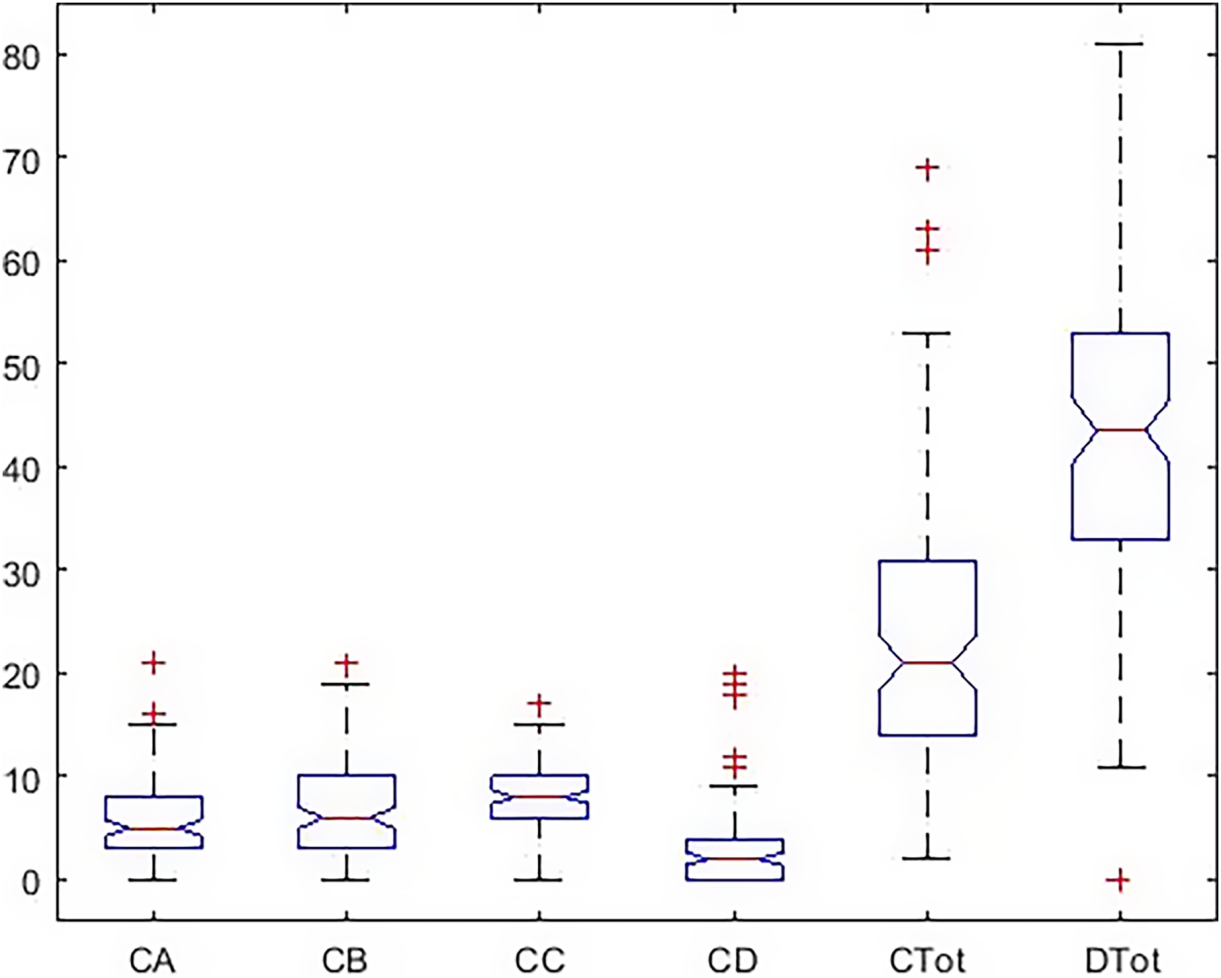
Boxplot of all indicators in a total statistical population.

**Figure 3:**
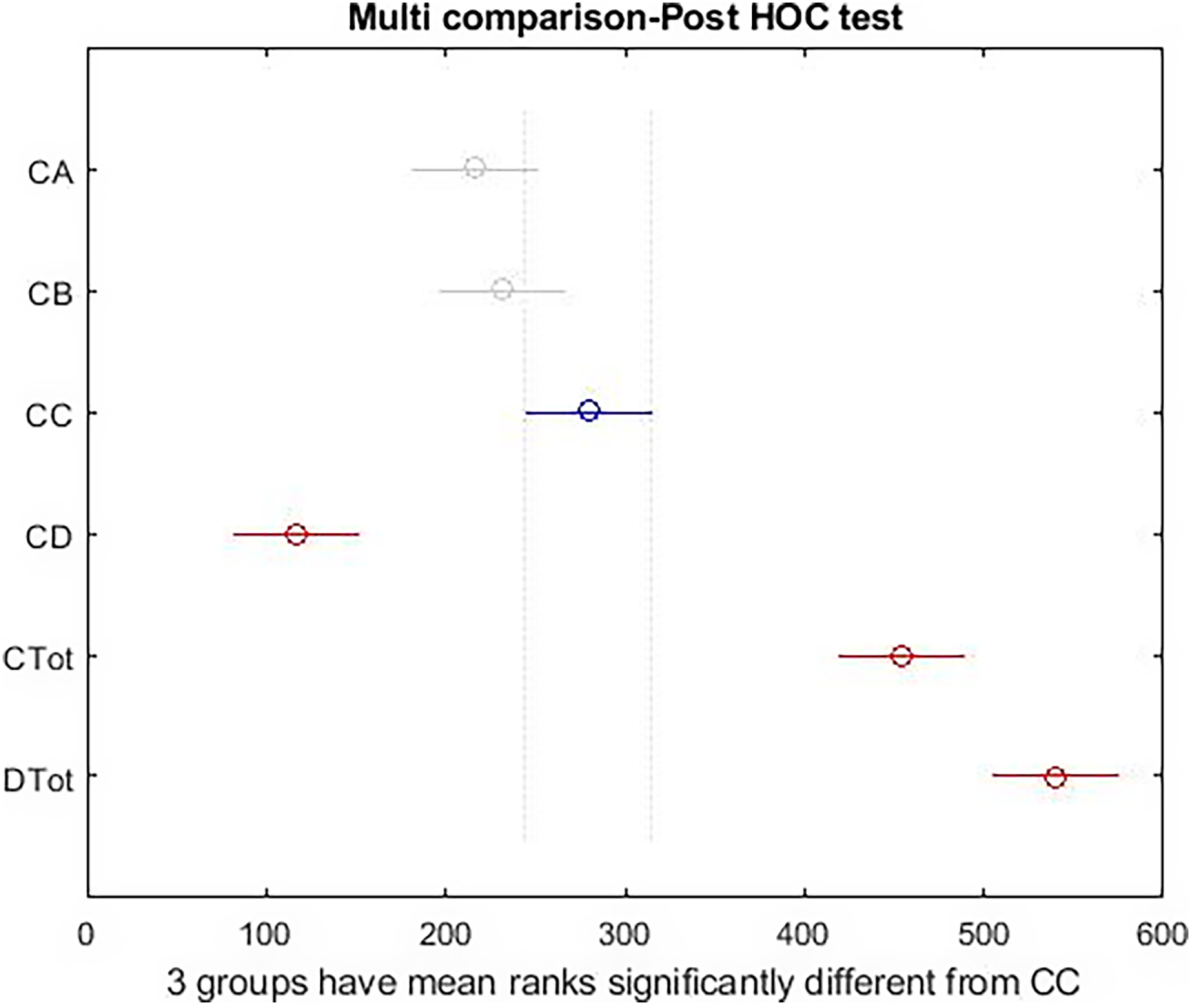
*Post hoc* test on Kruskal–Wallis test results.

There was a significant difference among all subscales and total health and happiness. Results are shown in Table 3. Also, Figs. 2 and 3 report the results of the Tukey Test.

**Table 3:** Results for all indicators of health and happiness by the Kruskal–Wallis test.

Variable	Total squares	Mean squares	Chi-SQ	Probchi-SQ
Indexes	1.294 * 10^7^	2,569,067.5	414.95	
Error	6.116 * 10^6^	10,092.6		1.774 * 10^−87^
Total	1.906 * 10^7^			

The graphical form of the distribution is shown in Fig. 3; the high differences between the right side indicators of the box diagram with the health measures reported by the Kruskal–Wallis test. As shown in Fig. 3, there is a significant difference among total health and happiness and all other indices. Among sub-scales, depression differs significantly from the other three factors. The blue color represents the selected indicator compared with the rest. The gray color indicates no significant relationship between the selected indicator and the gray indices. The red color indicates a significant difference between the selected and red indices. According to the results obtained above and the reported differences between the females and males, the tests used for the entire community were made for the two groups to show the significance of differences between different indices in the two groups separately.

The analysis of the variance indicates a significant difference between all indices of study in boys (Table 4), according to the reported probability. In the following, the graphic representations of different indices are investigated, and then the follow-up tests are used to determine the significance of the difference between indicators.

**Table 4:** Kruskal–Wallis test in males for all indicators of health and happiness.

Variable	Total squares	Mean squares	Chi-SQ	Probchi-SQ
Indexes	1,053,412.9	210,682.6	181.11	
Error	476,307.1	1,846.2		3.100 * 10^−37^
Total	1,529,720			

**Figure 4:**
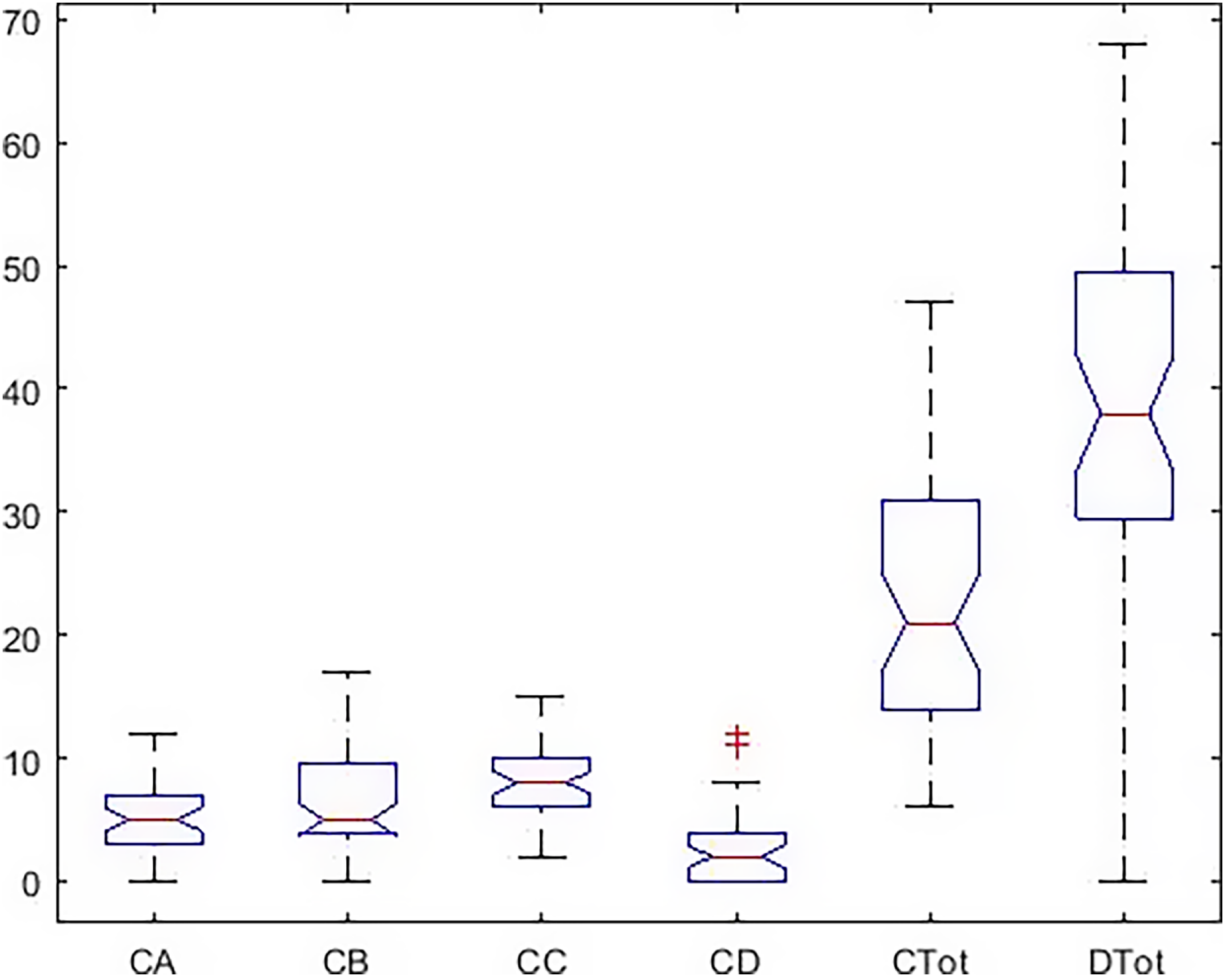
Boxplot of all indicators in the males.

In Fig. 4, the dispersion of the data, the standard deviation, the median, and the upper and low quartile are represented. According to the scoring of happiness questionnaire and its difference with a health questionnaire, the length of the box diagram of both the general health indices (Ctot) and happiness (Dtot) is well demonstrated.

**Figure 5:**
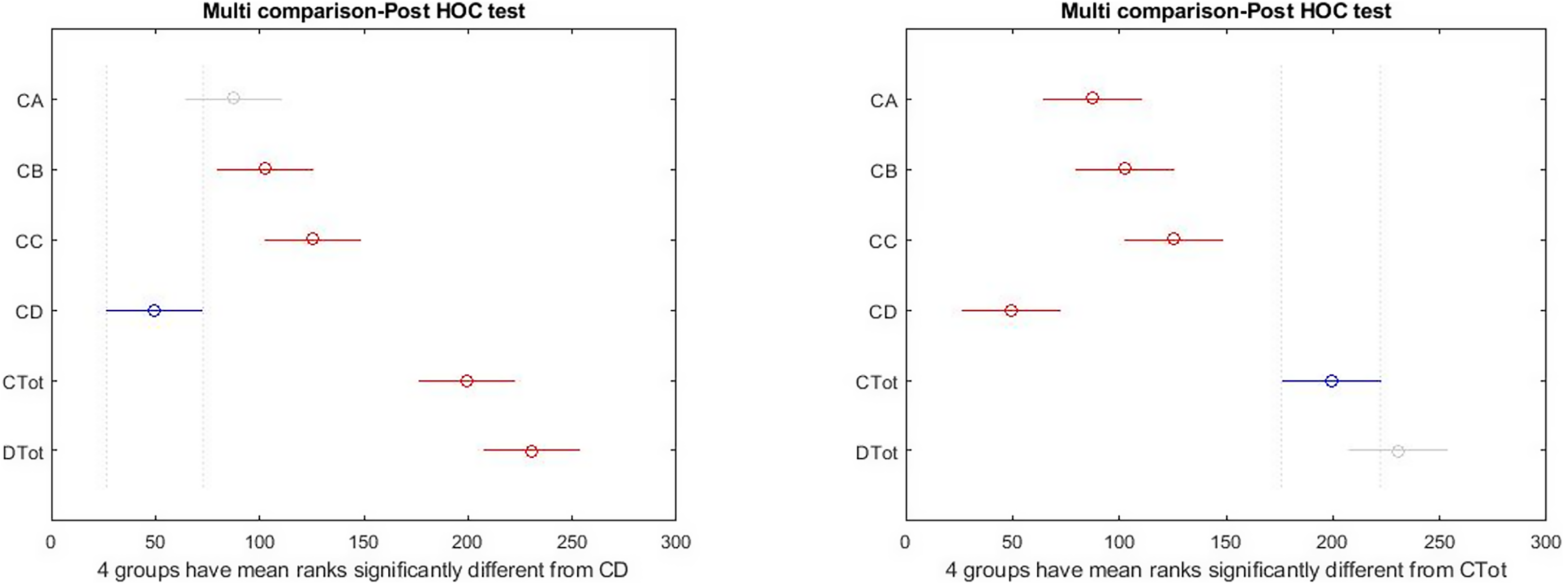
*Post hoc* test on Kruskal–Wallis test results in males.

The *post hoc* test results (Fig. 5) show a significant difference between total health and happiness in the boys’ group with all other indicators. Also, depression has a significant difference with anxiety and social function, but there is no significant difference with the physical dimension.

There was a significant difference between all indices (Table 5) in the girls’ group.

**Table 5:** Kruskal–Wallis test in males for all indicators of health and happiness.

Variable	Total squares	Mean squares	Chi-SQ	Probchi-SQ
Indexes	2.367 * 10^6^	473,535.9	181.11	
Error	1.136 * 10^6^	3,323.2		1.185 * 10^−48^
Total	3.504 * 10^6^			

**Figure 6:**
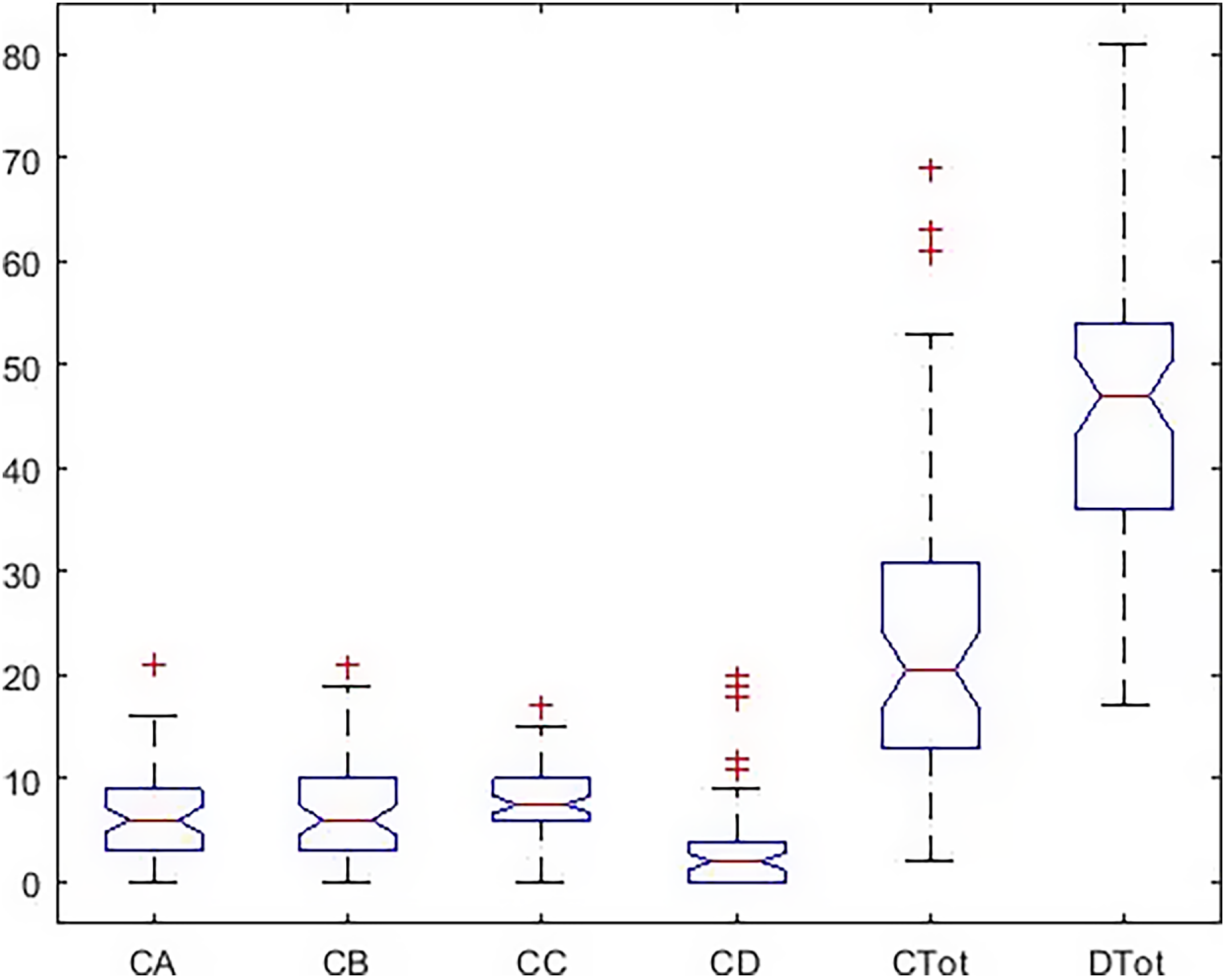
Boxplot of all indicators in the females.

Figure 6 shows that in the girls’ group, the dispersion of the data is higher. The amount of data outside a higher quartile is seen, representing deviations from the higher criteria reported in Table 1.

**Figure 7:**
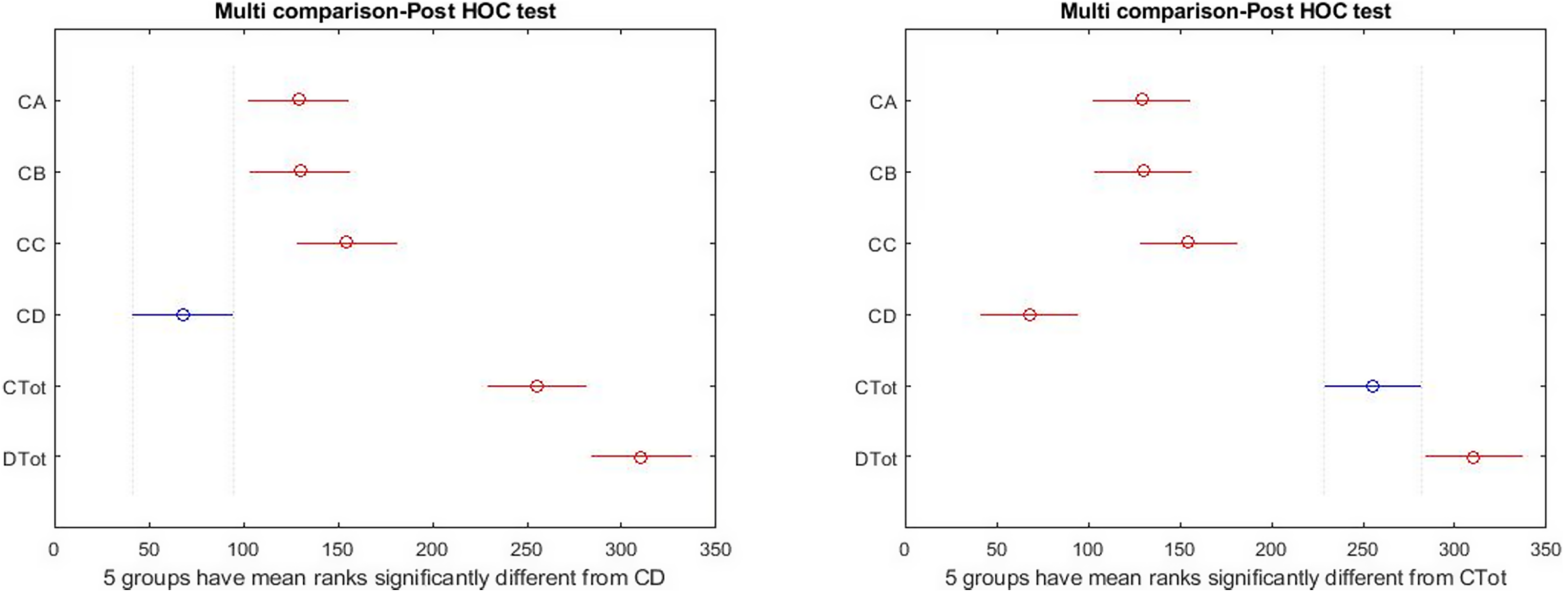
*Post hoc* test on Kruskal–Wallis test results in females.

The results of the Tukey–Kramer showed (Fig. 7) that there was a significant difference between total health and happiness with all other indices in the girls’ group. Also, according to the LSD test, there is a significant difference between depression and three dimensions of health scales. That is different from the results of the boys’ group.

**Figure 8:**
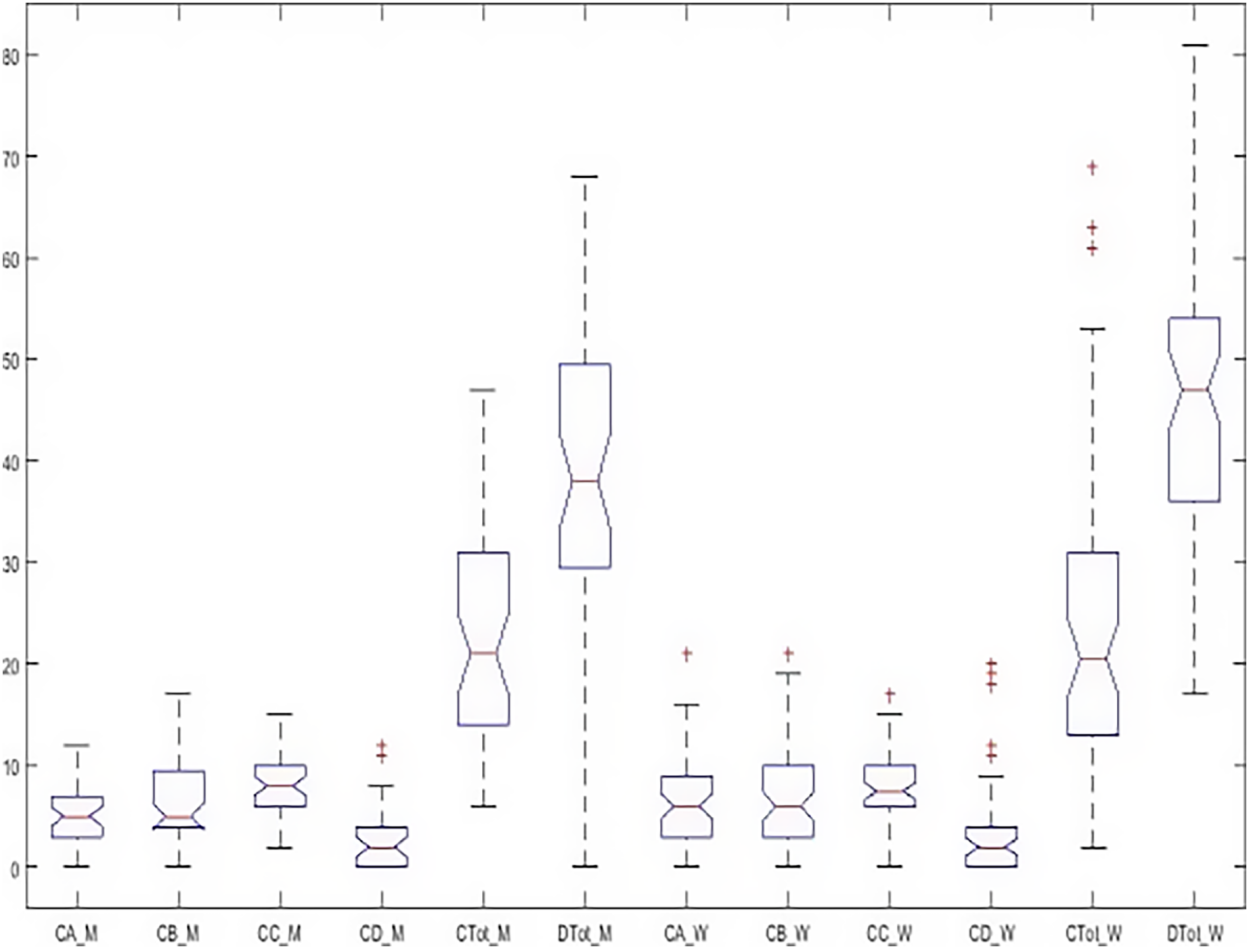
Boxplot of all indicators simultaneously in both groups.

**Figure 9:**
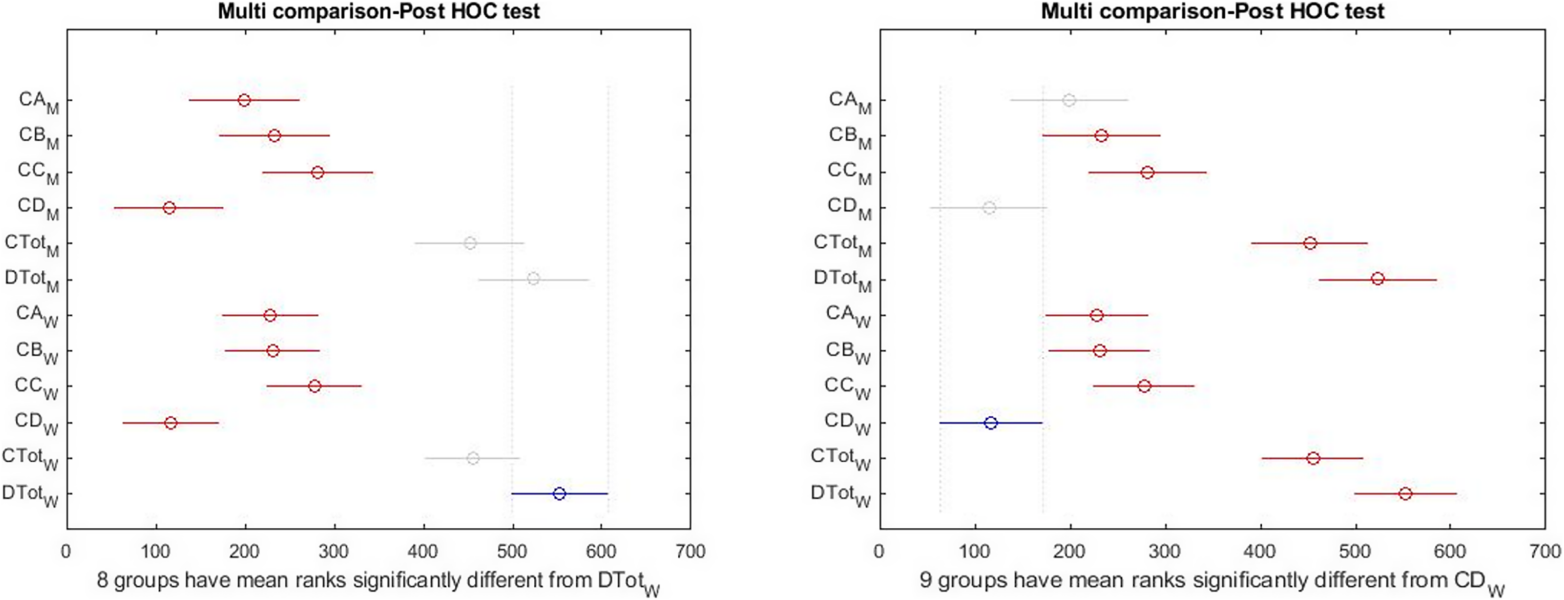
*Post hoc* test on Kruskal–Wallis test results simultaneously in both groups.

Figure 8 shows the dispersion of all the indices simultaneously and graphically in both groups. In this figure, the happiness in the girls’ group ( $Dtot_W$) is different from the boys ( $Dtot_M$) in terms of elongation, mean, and median. In order to explain the significance of these differences between all indicators, it was tested by Wallis and Tukey HUK and LSD, whose results are shown in Fig. 9.

Figure 9 (left) shows that health and happiness significantly differed with sub-scales. By changing the selected index from females’ happiness to females’ depression (blue color), the right figure shows that depression in the girls’ group is significantly different from all indices. Still, no significant difference between depression and physical symptoms was observed in boys. This means that despite differences, the level of depression in girls and boys is not significant.

**Figure 10:**
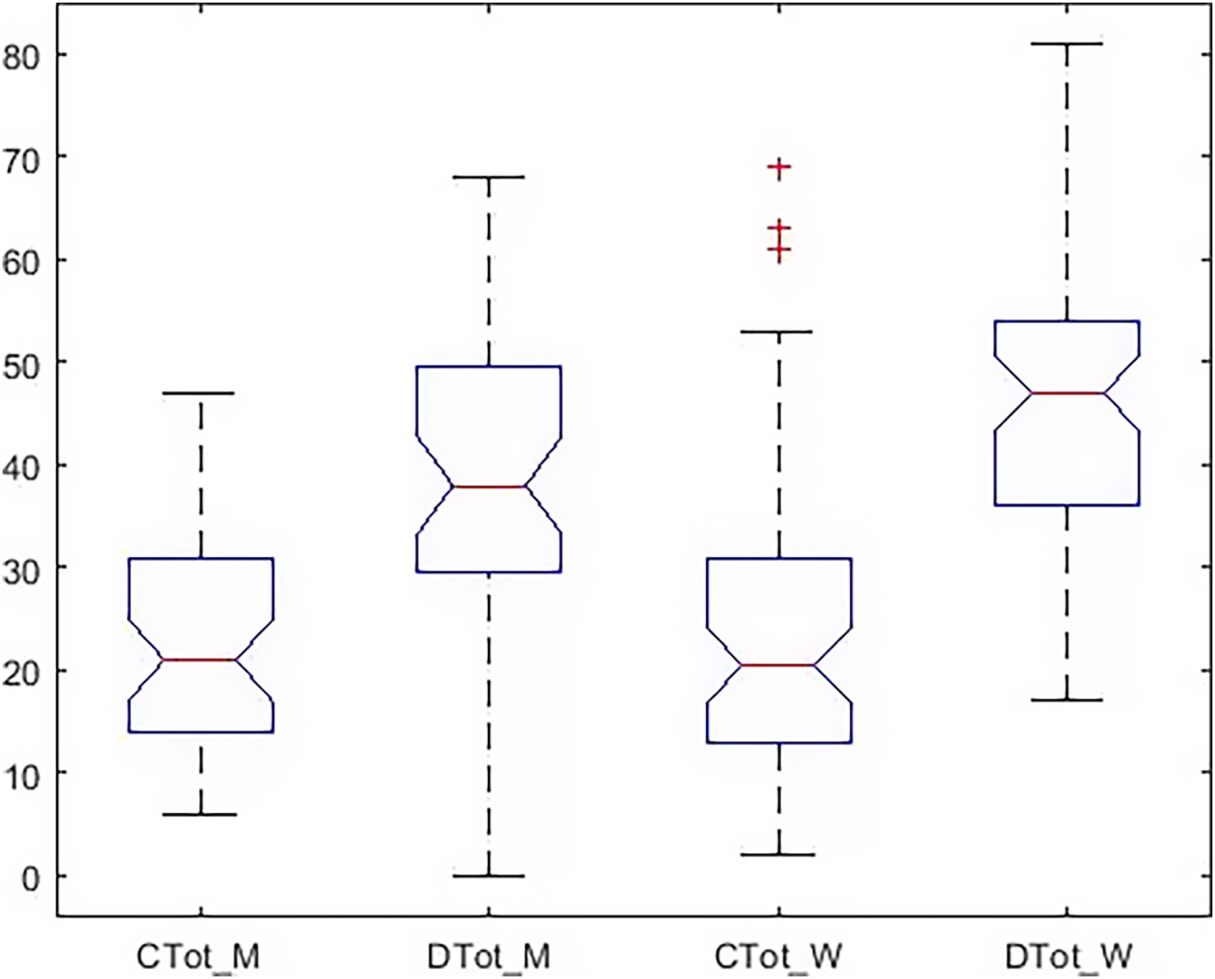
Boxplot of total health and happiness simultaneously in both group.

**Figure 11:**
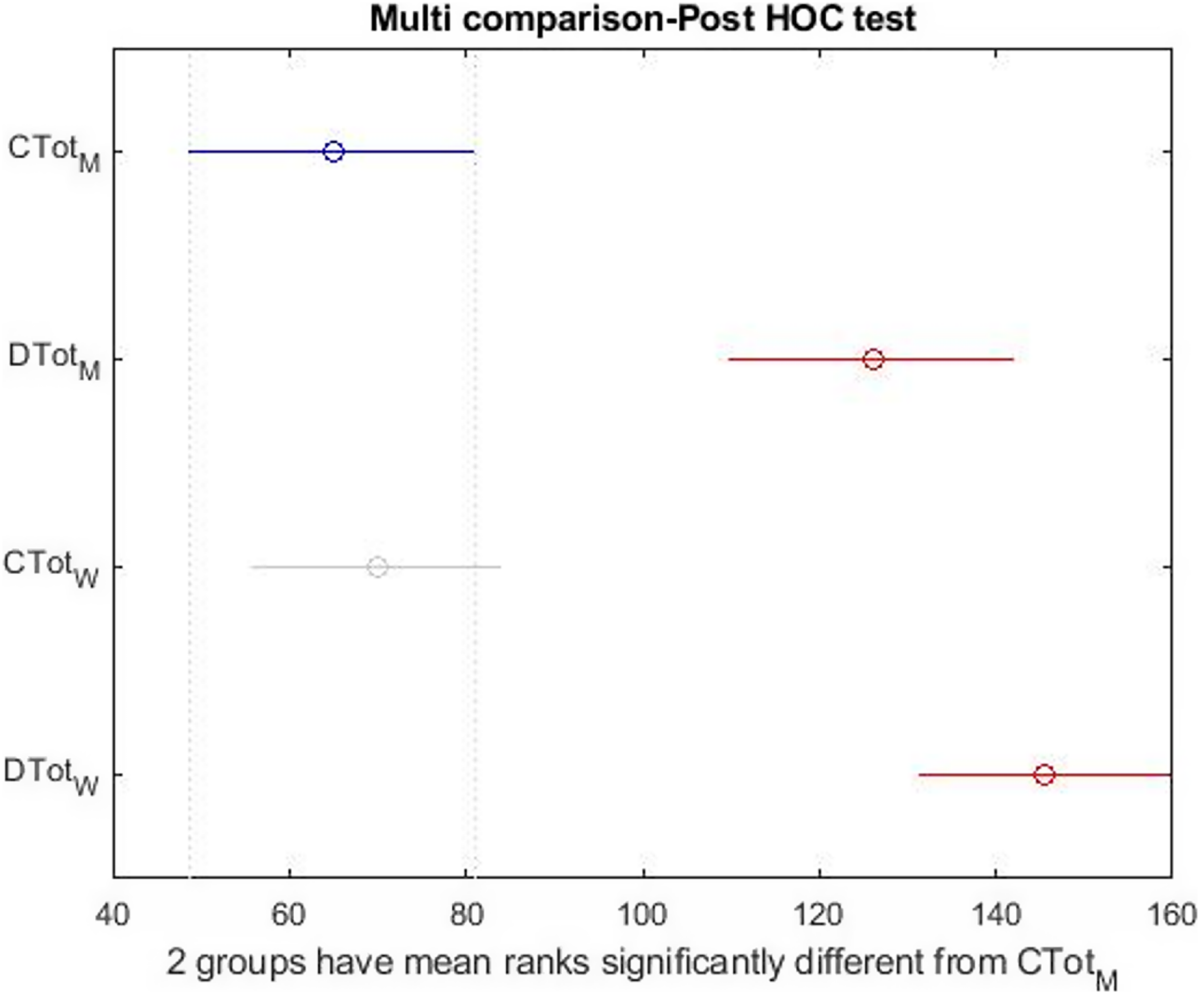
*Post hoc* test on the Kruskal–Wallis test results simultaneously in both group.

Figure 10 shows that the dispersion of health indicators is similar, but the dispersion of happiness is different in the two groups. In Fig. 11, there is a significant difference between subscales and happiness in both groups. So, differences in health indicators with each other and happiness must be evaluated. Finally, the relationship between the two indices was investigated independently. The Wilcoxon test was used to measure the correlation of the two indices with the unnormal distribution. Table 6 shows a significant difference between males’ and females’ happiness on the 5% level. In comparison, there is no significant difference between the health of the two groups. In Table 7, the relationship of subscales together in both groups is reported separately. According to Table 7, despite the differences reported above, none of the sub-scales significantly differ between the girls and boys at the 5% level. In other words, when subscales were separately evaluated, significant differences occurred only within the girls’ and the boys’ groups, not between groups.

**Table 6:** The relationship between total health and happiness among two groups by Wilcoxon test.

Health–Happiness	*P*	Z value	Rank sum
Health	0.9784	0.0270	2.27 * 10^3^
Happiness	0.043	−2.0	1.97 * 10^3^

**Table 7:** The relationship between subscales in two groups by Wilcoxon test.

Subscales	*P*	Z value	Rank sum
Physical symptoms (CA)	0.3403	−0.9536	2,125
Anxiety (CB)	0.9083	0.1152	2.28 * 10^3^
Social functioning (CC)	0.8195	0.2282	2,300
Depression (CD)	0.7720	0.2898	2.30 * 10^3^

## Discussion

Many studies have examined the relationship between happiness and health. However, few studies have investigated happiness and health in both male and female students. This study showed the means of subscales in males (physical symptoms = $5.22 \pm 2.92$, anxiety = 6.63 $\pm$ 4.26, social function = 7.97 $\pm$ 3.06 and depression = 2.77 $\pm$ 3.19) and in females (physical symptoms = 6.62 $\pm$ 4.74, anxiety = 6.91 $\pm$ 5.37, social function = 7.94 $\pm$ 3.48 and depression = 3.15 $\pm$ 4.67). In other words, males had better physical function and less anxiety and depression than females. According to the Kruskal–Wallis and Wilcoxon’s signed rank tests, there was a significant difference between total health and its subscales and happiness in both genders. In males, depression had a significant difference with anxiety and social function but in females with all three subscales (anxiety, social function, and physical function). The happiness mean was higher in females (45.73 $\pm$ 14.64) than males (39.06 $\pm$ 14.37). Concerning the relationship between happiness and gender, the present study results are consistent with the findings of previous studies on university students *e.g*. Farhadi et al. (2005) and Sharifi et al. (2010). However, they are contrary to the findings of some other studies for example, Alavi (2007), Koivumaa-Honkanen et al. (2005), Siamian et al. (2012), Rafiei, Mosavipour & Aghanajafi (2012), and Rajabi, Saremi & Bayazi (2012). Diener, Oishi & Lucas (2003) and Fujita, Diener & Sandvik (1991) found almost no difference between males and females, while in the present study, females are happier than males. This discrepancy may be explained by the fact that women are more likely to express their emotions in social relationships. Nevertheless, things get a little complicated regarding depression and anxiety. Despite more happiness in females, depression and anxiety also had a higher average than males. It was also explained that women experience both more negative and positive emotions (Diener & Biswas-Diener, 2002) and report more negative emotions and depression. They are more likely to pursue these differences than men. Probably women are more likely to be more receptive to their negative emotions, while men deny having such factors (Eddington & Shuman, 2005). Thus, depression findings of more negative effect in women do not conflict with well-being findings of equal happiness across gender. Generally, women’s more intense positive emotions balance their higher negative effects (Fujita, Diener & Sandvik, 1991). On the other hand, the sources of happiness are different for the two genders. Men are more affected by themselves, their jobs, and economic satisfaction, and women more by their children and family health. Another factor expressing this difference is probably the way people think and behave, which can determine their happiness. Therefore, maybe Students who reported more happiness tended to improve their healthy lifestyles more than the other groups. Francis et al. (1998) calculated the mean of happiness in boys and girls in Canada, the United States, England, and Australia, 42 and 32, respectively. In the present study, this means was obtained (45.73 $\pm$ 14.64 and 39.06 $\pm$ 14.37) greater for females and less for males than those countries. Comparison of the means of total health and happiness in the present study with other research shows that some of them reported a higher mean (Jafari, Liaghatdar & Abedi, 2004; Yazde Khasti, Ahmadi Forooshani & Arizi, 2015), and some other studies a lower mean (Rafiei, Mosavipour & Aghanajafi, 2012). In explaining these findings, it can be argued that human beings usually seek happiness, and happiness is especially important in people’s lives, while the experience of stress and anxiety significantly reduces the feeling of happiness. Sarb, Cimpean & Grigoras (2010) reported that girls had more disorders (Üner et al., 2008), but the results of the study by Solgi, Saeedipoor & Abdolmaleki (2009) show that the rate of disorder in both genders was almost equal. In the present study, there was a strong correlation between happiness and total health and anxiety and the weakest association with physical symptoms. Also, the correlation between happiness and all indicators except social symptoms in females was more than males. At the same time, only the association between physical symptoms and happiness has been reported high in males than females. The most important factor associated with happiness in females was depression and anxiety, but it was contrary for males. This shows the profound effect of depression on females’ happiness. Another interesting point reported is the difference between the performances of the physical symptoms between the two groups. Physical symptoms had the least possible relationship with the social function in males and with depression in females. In general, it can be stated that the interaction of health indicators in females is completely different from males. Therefore, the effect of different indicators on each other has been reported tangible in females. In other words, males had better health symptoms and less depression and anxiety than females, but they had similar and weak social functions. In general, it can be argued that by promoting general health, the levels of general self-efficacy and happiness rise. General health is one of the factors whose relationship with happiness can affect the representation of the dimensions of happiness. In fact, by increasing happiness, general health rises, and life gets more pleasant. Also, a better ground is prepared for people’s growth and development in different areas, such as more effective and efficient interpersonal relationships. This was one of the problematic areas for the present study participants (the mean score of the social functioning index). The cross-sectional nature of the information used to analyze the research findings makes it impossible to infer and understand the causal relationship between happiness and health in the two groups of males and females. Despite this limitation, this study has significant strengths. Participants of this study were a representative sample of students that enables generalizability. This study analyzed the relationship between health and happiness and compared male and female students. The findings of this study show that happiness and health are significantly different between male and female students. Future research is needed to identify factors affecting the promotion of happiness and public health among students to inform policymakers and public health officials, youth affairs planners, university officials, and those involved in student affairs in this regard.

## Acknowledgements

The author would like to express gratitude to the four anonymous reviewers for their useful comments and editorial suggestions, which improved the comprehension of the manuscript. The author would like to thank the participants of the study for their time and participation.

## Supplemental Information

10.7717/peerj.14339/supp-1 Supplemental Information 1 Matlab Script.

10.7717/peerj.14339/supp-2 Supplemental Information 2 OHI.

## Additional Information and Declarations

### Data Availability

The following information was supplied regarding data availability: The script is available in the Supplemental File.

## References

[ref-1] Alavi HR (2007). Correlatives of happiness in the university students of Iran (a religious approach). Journal of Religion and Health.

[ref-3] Diener E, Biswas-Diener R (2002). Will money increase subjective well-being?. Social Indicators Research.

[ref-4] Diener E, Oishi S, Lucas RE (2003). Personality, culture, and subjective well-being: emotional and cognitive evaluations of life. Annual Review of Psychology.

[ref-5] DiMaria CH, Peroni C, Sarracino F (2020). Happiness matters: productivity gains from subjective well-being. Journal of Happiness Studies.

[ref-6] Dolan P (2014). Happiness by design: change what you do, not how you think.

[ref-7] Eddington N, Shuman R (2005). Subjective well being (happiness). https://www.texcpe.com/html/pdf/ca/ca-happiness.pdf.

[ref-8] Farhadi A, Javaheri F, Gholami YB, Farhadi P (2005). The amount of mirthfulness and its relation with self-reliance in students of Lorestan university of medical sciences. https://www.sid.ir/paper/88532/en.

[ref-9] Francis LJ, Brown LB, Lester D, Philipchalk R (1998). Happiness as stable extraversion: a cross-cultural examination of the reliability and validity of the Oxford Happiness Inventory among students in the UK, USA, Australia, and Canada. Personality and Individual Differences.

[ref-10] Francis LJ, Ziebertz H-G, Lewis CA (2003). The relationship between religion and happiness among German students. Pastoral Psychology.

[ref-11] Fujita F, Diener E, Sandvik E (1991). Gender differences in negative affect and well-being: the case for emotional intensity. Journal of Personality and Social Psychology.

[ref-12] Graham C (2012). Happiness around the world: the paradox of happy peasants and miserable millionaires.

[ref-13] Jafari E, Liaghatdar M, Abedi M (2004). Happiness and its degree of effective factors in students of the Isfahan Medical Sciences University. Journal of Fundamentals of Mental Health.

[ref-14] Kamthan S, Sharma S, Bansal R, Pant B, Saxena P, Chansoria S, Shukla A (2019). Happiness among second year MBBS students and its correlates using Oxford Happiness Questionnaire. Journal of Oral Biology and Craniofacial Research.

[ref-15] Koivumaa-Honkanen H, Kaprio J, Honkanen RJ, Viinamäki H, Koskenvuo M (2005). The stability of life satisfaction in a 15-year follow-up of adult Finns healthy at baseline. BMC Psychiatry.

[ref-16] Layard PR, Layard R (2011). Happiness: lessons from a new science.

[ref-17] Lester PB, Stewart EP, Vie LL, Bonett DG, Seligman ME, Diener E (2022). Happy soldiers are highest performers. Journal of Happiness Studies.

[ref-18] Lyubomirsky S, King L, Diener E (2005). The benefits of frequent positive affect: does happiness lead to success?. Psychological Bulletin.

[ref-19] Meisenberg G, Woodley MA (2015). Gender differences in subjective well-being and their relationships with gender equality. Journal of Happiness Studies.

[ref-20] Noddings N (2003). Happiness and Education.

[ref-21] Peltzer K, Pengpid S, Sodi T, Mantilla Toloza SC (2017). Happiness and health behaviours among university students from 24 low, middle and high income countries. Journal of Psychology in Africa.

[ref-22] Rafiei M, Mosavipour S, Aghanajafi M (2012). Happiness, mental health, and their relationship among the students at Arak University of Medical Sciences in 2010. Journal of Arak University of Medical Sciences.

[ref-23] Rajabi M, Saremi AA, Bayazi MH (2012). The relationship between religious coping patterns, mental health and happiness. Journal of Iranian Psychologists.

[ref-24] Sachs JD, Layard R, Helliwell JF (2018). World happiness report 2018. https://worldhappiness.report/ed/2018/.

[ref-25] Sarb S, Cimpean AM, Grigoras D (2010). Tie2 expression in human embryonic tissues. Romanian Journal of Morphology and Embryology.

[ref-26] Shamshiri B, Dastouri N (2018). Report of happiness in the Iranian educational system: a qualitative research. International Journal of Educational and Pedagogical Sciences.

[ref-27] Sharifi K, Souki Z, Tagharobi Z, Akbari H (2010). Happiness and its related factors among the students of Kashan University of Medical Sciences in 2006-7. Feyz.

[ref-28] Siamian H, Naeimi OB, Shahrabi A, Hassanzadeh R, Abazari MR, Khademloo M, Javadian Koutenaee M (2012). The status of happiness and its association with demographic variables among the paramedical students. Journal of Mazandaran University of Medical Sciences.

[ref-29] Solgi Z, Saeedipoor B, Abdolmaleki P (2009). Study of psychological well-being of physical education students of Razi university of Kermanshah. Journal of Kermanshah University of Medical Sciences.

[ref-30] Steptoe A (2019). Happiness and health. Annual Review of Public Health.

[ref-32] Üner S, Özcebe H, Telatar TG, Tezcan S (2008). Assessment of mental health of university students with GHQ-12. Turkish Journal of Medical Sciences.

[ref-31] Yazde Khasti F, Ahmadi Forooshani HA, Arizi HR (2015). The causal model of relationship between religious attitude, happiness, pleasure and mental health in students. Journal of Educational Psychology Studies.

