# Supplementary material for: Gender differences in general health and happiness: a study on Iranian engineering students
Source: PeerJ. 2022 Nov 3;10:e14339. doi: 10.7717/peerj.14339 (PMC9637358; doi:10.7717/peerj.14339)
Supplement: Supplemental Information 2 [file peerj-10-14339-s002.pdf]

Oxford Happiness Questionnaire

The Oxford Happiness Questionnaire was developed by psychologists Michael Argyle and Peter Hills at Oxford University.

Instructions

Below are a number of statements about happiness. Please indicate how much you agree or disagree with each by entering a number in the blank after each statement, according to the following scale:

1 = strongly disagree
2 = moderately disagree
3 = slightly disagree
4 = slightly agree
5 = moderately agree
6 = strongly agree

Please read the statements carefully, some of the questions are phrased positively and others negatively. Don't take too long over individual questions; there are no "right" or "wrong" answers (and no trick questions). The first answer that comes into your head is probably the right one for you. If you find some of the questions difficult, please give the answer that is true for you in general or for most of the time.

The Questionnaire

1. I don't feel particularly pleased with the way I am. (R) _
2. I am intensely interested in other people. _
3. I feel that life is very rewarding. _
4. I have very warm feelings towards almost everyone. _
5. I rarely wake up feeling rested. (R) _
6. I am not particularly optimistic about the future. (R) _
7. I find most things amusing. _
8. I am always committed and involved. _
9. Life is good. _
10. I do not think that the world is a good place. (R) __
11. I laugh a lot. _
12. I am well satisfied about everything in my life. _
13. I don't think I look attractive. (R) _
14. There is a gap between what I would like to do and what I have done. (R) _
15. I am very happy. _

16. I find beauty in some things. _
17. I always have a cheerful effect on others. _
18. I can fit in (find time for) everything I want to. _
19. I feel that I am not especially in control of my life. (R) _
20. I feel able to take anything on. _
21. I feel fully mentally alert. _
22. I often experience joy and elation. _
23. I don't find it easy to make decisions. (R) _
24. I don't have a particular sense of meaning and purpose in my life. (R) _
25. I feel I have a great deal of energy. _
26. I usually have a good influence on events. _
27. I don't have fun with other people. (R) _
28. I don't feel particularly healthy. (R) _
29. I don't have particularly happy memories of the past. (R) _

Calculate your score

Step 1. Items marked (R) should be scored in reverse:

For example, if you gave yourself a "1," cross it out and change it to a "6."
Change "2″ to a "5″
Change "3″ to a "4″
Change "4″ to a "3″
Change "5″ to a "2″
Change "6″ to a "1″

Step 2. Add the numbers for all 29 questions. (Use the converted numbers for the 12 items that are reverse scored.)

Step 3. Divide by 29. So your happiness score = the total (from step 2) divided by 29.

Your Happiness Score:

Interpreting the score, by Stephen Wright

1-2: Not happy. If you answered honestly and got a very low score, you're probably seeing yourself and your situation as worse than it really is. I recommend taking the Depression Symptoms test (CES-D Questionnaire) at the University of Pennsylvania's "Authentic Happiness" Testing Center. You'll have to register, but this is beneficial because there are a lot of good tests there and you can re-take them later and compare your scores.

2-3: Somewhat unhappy. Try some of the exercises on this site like the Gratitude Journal & Gratitude Lists, or the Gratitude Visit; or take a look at the "Authentic Happiness" site mentioned immediately above.

3-4: Not particularly happy or unhappy. A score of 3.5 would be an exact numerical average of happy and unhappy responses. Some of the exercises mentioned just above have been tested in scientific studies and have been shown to make people lastingly happier.

4: Somewhat happy or moderately happy. Satisfied. This is what the average person scores.

4-5: Rather happy; pretty happy. Check other score ranges for some of my suggestions.

5-6: Very happy. Being happy has more benefits than just feeling good. It's correlated with benefits like health, better marriages, and attaining your goals. Check back – I'll be writing a post about this topic soon.

6: Too happy. Yes, you read that right. Recent research seems to show that there's an optimal level of happiness for things like doing well at work or school, or for being healthy, and that being "too happy" may be associated with lower levels of such things.

Reference

Hills, P., & Argyle, M. (2002). The Oxford Happiness Questionnaire: a compact scale for the measurement of psychological well-being. *Personality and Individual Differences*, 33, 1073–1082.

Stephen Wright is a visiting scientist at Georgetown University's Brain and Language Lab, and founder of meaningandhappiness.com

Reference:
http://www.meaningandhappiness.com/oxford-happiness-questionnaire/214/
